# Inferring 3D chromatin structure using a multiscale approach based on quaternions

**DOI:** 10.1186/s12859-015-0667-0

**Published:** 2015-07-29

**Authors:** Claudia Caudai, Emanuele Salerno, Monica Zoppè, Anna Tonazzini

**Affiliations:** 1 National Research Council of Italy, Institute of Information Science and Technologies, Via Moruzzi, 1, Pisa, 56124 Italy; 2 National Research Council of Italy, Institute of Clinical Physiology, Via Moruzzi, 1, 56124 Pisa, Italy

**Keywords:** 3d chromatin structure, Chromosome conformation capture, Multiscale approach, Quaternions

## Abstract

**Background:**

The knowledge of the spatial organisation of the chromatin fibre in cell nuclei helps researchers to understand the nuclear machinery that regulates dna activity. Recent experimental techniques of the type *Chromosome Conformation Capture* (3c, or similar) provide high-resolution, high-throughput data consisting in the number of times any possible pair of dna fragments is found to be in contact, in a certain population of cells. As these data carry information on the structure of the chromatin fibre, several attempts have been made to use them to obtain high-resolution 3d reconstructions of entire chromosomes, or even an entire genome. The techniques proposed treat the data in different ways, possibly exploiting physical-geometric chromatin models. One popular strategy is to transform contact data into Euclidean distances between pairs of fragments, and then solve a classical distance-to-geometry problem.

**Results:**

We developed and tested a reconstruction technique that does not require translating contacts into distances, thus avoiding a number of related drawbacks. Also, we introduce a geometrical chromatin chain model that allows us to include sound biochemical and biological constraints in the problem. This model can be scaled at different genomic resolutions, where the structures of the coarser models are influenced by the reconstructions at finer resolutions. The search in the solution space is then performed by a classical simulated annealing, where the model is evolved efficiently through quaternion operators. The presence of appropriate constraints permits the less reliable data to be overlooked, so the result is a set of plausible chromatin configurations compatible with both the data and the prior knowledge.

**Conclusions:**

To test our method, we obtained a number of 3d chromatin configurations from hi-c data available in the literature for the long arm of human chromosome 1, and validated their features against known properties of gene density and transcriptional activity. Our results are compatible with biological features not introduced *a priori* in the problem: structurally different regions in our reconstructions highly correlate with functionally different regions as known from literature and genomic repositories.

**Electronic supplementary material:**

The online version of this article (doi:10.1186/s12859-015-0667-0) contains supplementary material, which is available to authorized users.

## Background

The packing of DNA in living cells is obtained through several mechanisms, both general (due to general princples, irrespective of DNA sequence) and specific, *i.e.* mediated by proteins that recognise specific motifs and bring in close proximity parts of DNA that may be very distant in the genomic sequence. The first level, mediated by histone octamers, produces a fibre of about 11 nm. This fibre, in turn, is supposed to be organised into a 30 nm-wide structure, whose existence, however, is still debated [1, 2]. Most current information on packaging is derived from data that are not necessarily consistent with a single conformation, because they are obtained from a pool of cells which are not synchronized, even if they are of the same kind. As a result of the activities involving DNA (transcription, replication, repair, silencing etc.), in different individual cells, DNA organization can be slightly different, while responding to the same general principles. It is also to be kept in mind that DNA is not a rigid entity, and its structure changes from moment to moment in the same cell, both to respond to external stimuli (allowing for either transcription regulation or DNA repairs, if necessary), and to allow for regular compaction, as clearly recognisable at large scale during mitosis. It is well established that, in interphase cells, most chromosomal DNA is organised in ‘chromosome territories’ [3], and it is increasingly apparent that chromosomal organisation is one of the factors involved in regulation of gene function.

A step ahead towards an understanding of this spatial organisation has been enabled by fluorescence *in-situ* hybridisation techniques (FISH [4, 5]), which can be used to locate specific DNA sequences in the genome and measure the distances between pairs of fragments. More recently, Chromosome Conformation Capture (3C, [6]) and a number of related techniques (4C [7], 5C [8], Hi-C [9, 10]) fostered a major boost in chromatin studies, as they provide high-throughput, high-resolution contact data for a full genome at a relatively low cost. The output of each such experiment is a matrix of contact frequencies between pairs of DNA fragments in a uniform population of cells. The average size of the individual fragments depends on the restriction enzymes used. For example, the fragment sizes in the data we use here, obtained by enzyme HindIII, are of about 4 kbp. The raw contact matrices can thus have a very high genomic resolution, but the data come from millions of cells, so stable results can only be obtained by binning the matrices to lower resolutions (typically, 100 kbp). A new experimental protocol [11] applied to individual cells confirms the validity of Hi-C results, pointing out that the intra-chromosomal structures are substantially stable across different cells, whereas a marked variability of inter-chromosomal interactions has been revealed. Since Chromosome Conformation Capture data carry information about the 3D spatial configuration of the chromatin chain, many research groups in the last decade have been trying to develop specific reconstruction algorithms.

The earliest attempts in this sense used constrained optimisation techniques, mostly looking for an explicit and deterministic relationship between the contact frequencies and the Euclidean distances between pairs of fragments in the 3D conformation [6, 12–14]. The intuitive strength of this choice is that pairs of fragments that are frequently in contact are likely to be spatially close, whatever their genomic distance; vice versa, pairs of fragments with a few contacts are assumed to be farther apart. In [6], a theoretical expression for worm-like chains [15] is adopted, whereas [12] and [13], among others, assume some negative-power relationship between the distances and the contact frequencies. Other approaches include fitting an empirical distance-frequency law to FISH experimental data [16], and using a golden-section search to choose among a parametric family of relationships [14]. In [17], it is proposed to correlate contact frequencies with the presence or absence of chromatin contacts rather than with average distances. Once the distances between all the possible pairs of loci have been determined, the optimisation approaches estimate the best-fit 3D structure from different models, such as piecewise linear curves [12] and bead-chain models [13, 16, 18, 19], by also enforcing various constraints derived from known geometric and topological features of the chromatin fibre. In [13] and [16], the constraints are derived from polymer physics. Polymer models for the chromatin fibre have also been proposed in [20–24]. In [11], the 3D structure is obtained by restrained molecular dynamics simulations, at fine or coarse resolutions, where the restraints are flexible target distances derived from the Hi-C data. In [25–27], polymer models with no frequency-distance conversion are proposed, with different strategies to match the computed and measured contact frequencies.

Simple constrained optimisation in high-dimensional applications suffers from known drawbacks, such as trapping in local modes and unaccountability of biases. Moreover, without an explicit probabilistic model accounting for noise, the estimated structures might not be representative of statistically significant conformational features. This motivated the proposal of a number of probabilistic approaches, ranging from Markov Chain Monte Carlo sampling on an unconstrained fragment distribution [28] to a Bayesian approach with Poisson likelihood and uniform prior, also including known biases into the solution model [29, 30]. Again, assuming a deterministic frequency-distance relationship is a popular choice in these approaches. However, [31] proposes a method where distances and contacts are related probabilistically, through a Poisson distribution.

In our view, there are a number of drawbacks that must be overcome to get accurate and reliable 3D reconstructions of the chromatin structure. First of all, we share the concerns about the use of deterministic relationships between contact frequencies and Euclidean distances. If the original contact matrix has null elements, infinite mutual distances can only be avoided if sets of mutually adjacent fragments are binned together until the related contact matrix has all nonzero entries. This sets the genomic resolution achievable well below its theoretical possibilities. Moreover, we checked the topological consistency of the structures obtained from real data through the most popular frequency-distance relationships found in the literature [32] and, as already observed in [33], we found that the distances inferred are often severely incompatible with the Euclidean geometry. Translating contacts into distances is not appropriate for one more reason: two fragments often found in contact are likely to be spatially close in nearly all the configurations assumed by the chromatin, but the converse does not need to be true. Nothing says that two DNA fragments that are seldom in contact are also far from each other.

A second aspect to be considered is the use of a suitable chromatin model to constrain the solution. Enforcing a data fit with no constraint on the mutual positions of the fragments increases tremendously the domain of the feasible solutions, thus decreasing one’s confidence in their plausibility. In [30, 34] no geometric constraint is imposed on the solutions, and yet biologically plausible conformations are found. The price to be paid for this result is the large number of parameters to be estimated and the multiple heuristic sampling processes involved.

The approach we propose in this paper includes a constrained modified-bead-chain model and a Monte Carlo sampling on a likelihood function built directly from the contact data. This frees us from binning the matrix if not needed to stabilise the data, even though zero-valued entries are left, and avoids the solution of a distance-to-geometry problem based on inconsistent data. By direct inspection of the data structure, or from knowledge of confined domains that do not interact with other segments of the genome [27, 35], we can partition the data matrix so that each such domain can be reconstructed separately and then, recursively, lower the resolution to find the spatial relationships between larger and larger chromatin segments with fixed internal configurations. At each resolution considered, the contact matrix must be partitioned by direct inspection or other relevant knowledge. The spatial structure at the finest resolutions is then reconstructed assuming that the structure of each subchain is not modified by its interactions with the other domains. This allows us to choose the most appropriate resolution for each segment, thus attaining an accurate reconstruction at both local and global levels. To sample the solution space, the chain configuration is evolved by quaternions [36], which offer advantages over the popular rotation matrices using Euler angles. Indeed, altering the bead positions by quaternions is independent of Cartesian coordinates, maintains topological constraints, and is less expensive computationally: it only involves generating planar and dihedral angles and inter-bead distances. The only constraint that needs to be checked is related to spatial interferences between beads.

In what follows, we describe our approach, give details on our present algorithmic choices, and report on the results obtained from the data set provided in [9].

## Methods

### A multiscale modified bead-chain chromatin model

To build our chromatin model, we exploit the fact that the DNA sequences in some genomic regions show many internal contacts and very weak interactions with the rest of the genome [35]. This entails a contact frequency matrix with a number of diagonal blocks with relatively large entries, associated to row and column ranges whose entries are much smaller almost anywhere else. Each such block lists the number of mutual contacts of the restriction fragments within one of the above-mentioned regions (called *topologically associating domains*, or TADs), whose 3D configuration does not depend on the rest of the sequence, and can thus be reconstructed from the data in the related diagonal block alone. The spatial relationships among different TADs depend on the data outside the diagonal blocks. To account for such a lower resolution structure, we consider each TAD as a single locus, and bin the contact matrix so that it corresponds to a single entry. Then, a new block structure can be identified and estimated. This procedure can be repeated recursively until the lowest significant resolution is reached. The result is a chromatin model whose structure can be represented at multiple resolutions.

**Fig. 1:**
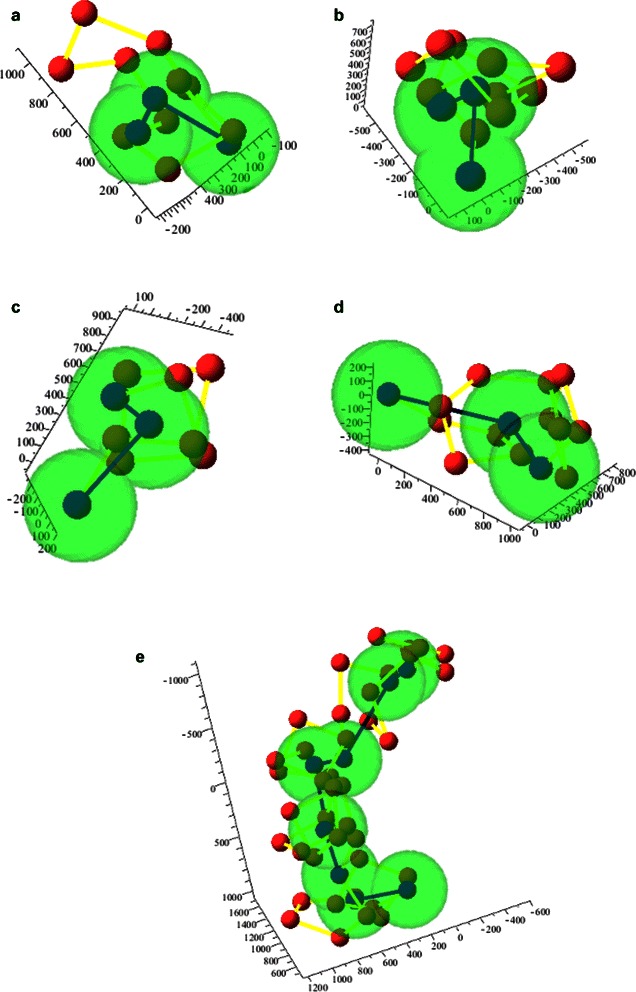
Modified bead-chain model. **a**–**d** Consecutive fragments of the chromatin fibre, represented as bead sequences (red balls linked by yellow segments), and as centroid-endpoints triples (blue balls linked by blue segments). The green spheres represent the assumed sizes for the beads at the lower resolution. **e** Lower-resolution chain composed by the fragments in **a**–**d**

We consider each locus, at any resolution, as a bead in a chain [37]. Given a chromatin fibre composed of subchains of known structures, we try to find their mutual positions, without changing their internal configurations, by modelling each of them through its geometric centroid, its start point, and its end-point. These three points and their mutual positions, associated with the estimated size of the subchain, constitute one bead of our model. The lengths of the segments joining the endpoints with the centroid, and the related angle, cannot be changed during the evolution of the model. Conversely, the planar and dihedral angles defining the position of each bead with respect to the adjacent ones can be varied, subject to possible constraints establishing flexibility and mutual distance ranges. The beads are linked in their biological order, with the end point of each bead coinciding with the start point of the next. Figure 1 illustrates how four consecutive subchains are schematised as modified beads and then connected to form a chain at a lower resolution. Of course, the structure of the fragments at the maximum allowed resolution is not known, so the centroid and the endpoints of each subchain collapse into a single point, that is, the beads become simple spheres.

The advantages offered by this model consist in a better accuracy in the reconstruction of the chain at successive resolutions. The lengths of the bonds linking each bead to its immediate neighbours are such that the beads cannot penetrate their neighbours and cannot be too far apart from them. The angles between adjacent bonds are constrained so that the chain curvature cannot be higher than biologically/physically permitted. Finally, the overall size of the chain in its 3D configuration cannot exceed the value of the size of the nucleus (*i*.*e*, 5 to 10 *μ*m). As opposed to what happens in [28] and [30], these constraints limit the feasible positions of any subset of loci, even though they do not affect the data fit term chosen to solve the reconstruction problem, as described in the next subsection.

### Contact frequency fit

We mentioned the difficulties arising when attempting to translate contact frequencies into distances, and the biases affecting the measured data [29, 38]. Our choice to build a data-fit criterion bypassing both these drawbacks consists in including the contact frequencies *n*_*i*,*j*_ from the contact matrix directly into the criterion. We assume that the bead pairs characterised by the largest contact numbers are likely to be in contact, whereas we do not say anything on the pairs with fewer contacts. The rationale for this choice is twofold: first, whatever their entity, the biases introduce the largest errors in the smallest contact frequencies; second, we do not try to enforce any target distance between pairs of beads. We just say that a pair must be in close contact, so we try to minimise its distance, subject to the constraints imposed on the whole chain, and weighted by the related contact frequency. In this way, the importance of any pair in the data fit is proportional to the contact frequency. In formulas, let $\mathcal {C}$ be the 3D configuration of the chromatin segment under study (a matrix containing the coordinates of all the bead centers), *d*_*i*,*j*_ be the Euclidean distance between the *i*-th and the *j*-th beads, and $\mathcal {L}$ be the set of pairs included in the data fit. We are free to exclude the pairs with low contact frequencies from $\mathcal {L}$, with the advantage of saving computation time. Our data fit term is

(1) $$ \Phi(\mathcal{C})=\sum_{i,j\in \mathcal{L}}n_{i,j}\cdot d_{i,j}   $$

where, if **x**_*i*_ and **x**_*j*_ identify two bead centers, it is *d*_*i*,*j*_=||**x**_*i*_−**x**_*j*_||; note that Eq. (1) does not imply any restriction on the contact matrix. Accepting a contact frequency to vanish simply means that the corresponding pair does not affect the data fit. Of course, all the configurations with *d*_*i*,*j*_ vanishing for each (*i*,*j*) in $\mathcal {L}$ are unconstrained minimisers of (1). Each such configuration has all the pairs of loci in $\mathcal {L}$ in contact, and all the others in arbitrary positions. This does not mean, however, that such configurations will all be reached: the geometrical constraints prevent the final structure from reaching all those minima, thus producing solutions that are consistent with both the data and our prior knowledge.

### Estimation strategy

#### Monte Carlo sampling

Let $\mathcal {C}$ be the configuration of a bead chain at any resolution. In our present implementation, we estimate it by sampling a probability density function $p(\mathcal {C})\propto \ \text {exp}[-\Phi (\mathcal {C})]$. The sampling is implemented by a Monte Carlo procedure with a classical annealing schedule [39, 40]. In synthesis, given the current chain configuration, a randomly altered configuration is proposed and included in the sample upon a probabilistic test. During the iteration, the data fit term $\Phi (\mathcal {C})$ is modified by dividing it by a decreasing *temperature* parameter, to make the distribution more peaked around its maxima. When the temperature has reached its minimum value, the samples generated should be clustered around the set of absolute maxima of the distribution. In our case, we expect that different configurations match the data equally well, so the distribution function is not expected to show very definite maxima. Thus, the simulated annealing is not used as a global optimiser: various configurations can show similar (low) values of the data fit and can be assumed as highly plausible solutions.

#### Model evolution: Quaternions

To evolve our model, we use quaternions rather than Euler angles (see Additional file 1, or reference [36] for a more complete account). Quaternions can represent very well rotations in a 3D space, as they are a simple framework to understand and visualise rotations using an angle and a rotation axis. Furthermore, quaternions avoid several problems involving rotations, such as singularities and numerical instabilities related to orthonormal matrices (*e.g.*, gimbal lock [41]). Finally, quaternions are less expensive than Euler angles, as they only need to store 4, as opposed to 9, real numbers, and composing two rotations needs 16 multiplications and 12 additions, as opposed to 27 multiplications and 18 additions.^1^

**Fig. 2:**
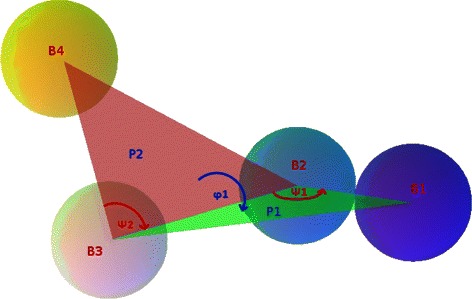
Quadruple of consecutive beads in a chain model. The two triples *B*_1_−*B*_2_−*B*_3_ and *B*_2_−*B*_3_−*B*_4_ determine, respectively, the planes *P*_1_ and *P*_2_ and the associated planar angles *ψ*_1_ and *ψ*_2_. Planes *P*_1_ and *P*_2_, in turn, determine the dihedral angle *φ*_1_

Quaternions are employed in many fields, including molecular dynamics and bioinformatics [42–44]. To see how their properties can be applied to perturb our model, let us consider the quadruple of consecutive beads in Fig. 2. Our model is a series of concatenated quadruples of this type. Once all the distances between the centers of consecutive beads and all the planar and dihedral angles are fixed, the position of each bead with respect to all the others is defined, and can easily be perturbed to obtain different chain conformations complying with the constraints. The planar angles are perturbed through simple quaternion operations by rotations around the normals to the corresponding planes, and the dihedral angles are perturbed by rotations around the intersection of the relevant planes. As an example, referring again to Fig. 2, a perturbation of angle *ψ*_1_ is obtained by rotating vector $\vec {B_{3}B_{2}}$ around the direction of the cross product between $\vec {B_{1}B_{2}}$ and $\vec {B_{3}B_{2}}$; a perturbation of angle *φ*_1_ is obtained by rotating vector $\vec {B_{4}B_{3}}$ around vector $\vec {B_{3}B_{2}}$. These operations maintain the chain topology, so the only constraint to be checked, if relevant, is the one that excludes spatial interference between beads.

### Overall recursive procedure

The recursive procedure we propose is described in this pseudocode:

structure = *procedure*(cont.matr, constraints)1) *e* *x* *t* *r* *a* *c* *t* *t* *h* *e* *d* *i* *a* *g* *o* *n* *a* *l* *b* *l* *o* *c* *k* *s* *f* *r* *o* *m* cont.matr2) For all the extracted blocks

a *Populate set* $\mathcal {L}$;
b *Set the initial bead chain configuration* $\mathcal {C}_{0}$;
c *Compute* $\Phi (\mathcal {C}_{0})$ *as in* Eq. (1);
d Iterate in *i* (assuming a cooling schedule *T*_0_→…
*T*_*n*_→…)
*Check stop criterion: if satisfied, save* $\mathcal {C}_{i}$ *and leave*
*Generate* $\mathcal {C}^{*}$ *by perturbing randomly the bond lengths, the planar and the dihedral angles of the current configuration* $\mathcal {C}_{i}$
*In the perturbed configuration, evaluate the distances between the beads belonging to the pairs in* $\mathcal {L}$;
*Compute* $\Phi (\mathcal {C}^{*})$
*if* {$\Phi (\mathcal {C}^{*}) < \Phi (\mathcal {C}_{i})$ *or* $\text {random}[0,1]< \mathrm {e}^{\left [\frac {\Phi (\mathcal {C}_{i}) - \Phi (\mathcal {C}^{*})}{T_{i}}\right ]}$}*and* constraints *are satisfied*
$\mathcal {C}_{i+1} = \mathcal {C}^{*}$
    else
$\mathcal {C}_{i+1} = \mathcal {C}_{i}$

3) *i* *f* *#* *o* *f* *d* *i* *a* *g* *o* *n* *a* *l* *b* *l* *o* *c* *k* *s*=1

structure = $\mathcal {C}$ (hierachical composition of all the saved configurations)

*output* structure

*leave*

4) constraints = *geometrical features of all the subchains + parameters and constraints at the new resolution* (Fig. 1 a-d)5) cont.matr = *bin*(cont.matr) (binning in accordance to the current blocks)6) structure= *procedure*(cont.matr, constraints)

We wrote Python 2.7.2 procedures implementing this recursion for two hierarchical levels (see Additional files 2, 3 and 4). At the highest resolution, we used external information on possible TADs to extract the diagonal blocks; further binnings should be based on the values assumed by the matrix elements, possibly using some appropriately chosen threshold. Note that step 2) can be performed in parallel for all the extracted blocks. This means that possible parallel computing capabilities can fully be exploited. Note also that this procedure produces *one* overall structure, at maximum resolution, per run. As per the remarks in the previous section, different runs normally produce different structures. Another way to proceed, for each data and parameter set, is to save all the stable subchain configurations at any resolution, and then sample each such set to produce the structures at the subsequent resolution. This strategy allows us to produce a potentially very large set of solutions, while saving much computation time. This is what we have done with the experiments reported below.

## Results and discussion

**Fig. 3:**
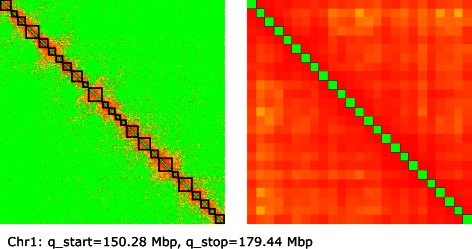
Experimental data. Heatmaps, in logarithmic scale, representing the contact matrix for the long arm of chromosome 1, from a Hi-C experiment on human lymphoblastoid cells (GM06990, [9]). Main diagonal removed for visualisation convenience. Left: Original 292×292 matrix at a resolution of 100 kbp. The 25 highlighted diagonal blocks represent the contacts in the topological domains used for binning. Right: 25×25 matrix obtained by binning the original

For our first experiments, we selected Hi-C data from the long arm of human chromosome 1 made available in [9]. The original resolution of these data was 100 kbp (Additional file 3). We partitioned these data with the help of the TADs identified in [35], thus obtaining 25 subchains of sizes ranging from 700 kbp to 1.8 Mbp (Additional file 4). After reconstructing the internal structures of these domains, we binned the data matrix so as to make a single entry from each of the blocks in the first partition, and run the algorithm again to estimate the entire chain at the new resolution. The structures of the original and the binned matrices are visualised in Fig. 3.

**Fig. 4:**
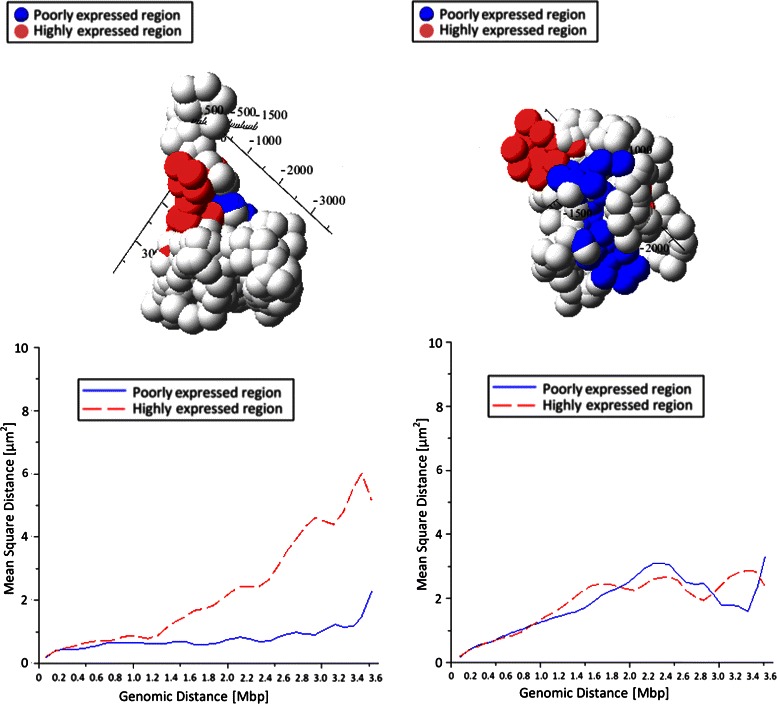
Example results. Top: the two typical configurations resulting from the data in Fig. 3 (measurements in nm). The model parameters used are: i) cardinality of sets $\mathcal {L}$ for the high-resolution subchains: 20; ii) cardinality of set $\mathcal {L}$ for the low-resolution chain: 40; iii) minimum distance between beads, at maximum resolution, to avoid interference: 120 nm; iv) maximum distance between any two beads: 10 *μ*m; v) maximum angle between two consecutive bead pairs (curvature): 100 °. The red and blue beads belong, respectively, to a highly expressed and a poorly expressed regions. Bottom: mean-square Euclidean distances between pairs of beads, as functions of their genomic distances, for the red and the blue regions

The starting size of each bead at each resolution was based on the number of internal contacts in the diagonal elements of the matrix. Intuitively, having many contacts between fragments belonging to the same locus means that the related DNA segment is very compact, so its size is small. Conversely, a few internal contacts mean a less packed locus, corresponding to a large bead in the chain. The other fundamental measures influencing our reconstructions are the lengths of the bonds between adjacent beads and the maximum planar angles described in Fig. 2. The lengths of the bonds have been derived from the sizes of the different beads, and are allowed to vary in specified ranges. The planar angles have been settled starting from biologically reasonable values considering the possible bending of the chromatin chain at each scale; then, the final values have been chosen on the basis of the overall size of the reconstructed chain, which must fit in the nucleus. This approach provides reconstructions that are already equipped with the appropriate measurements. The parameters used for all our experiments are reported in the caption to Fig. 4.

**Fig. 5:**
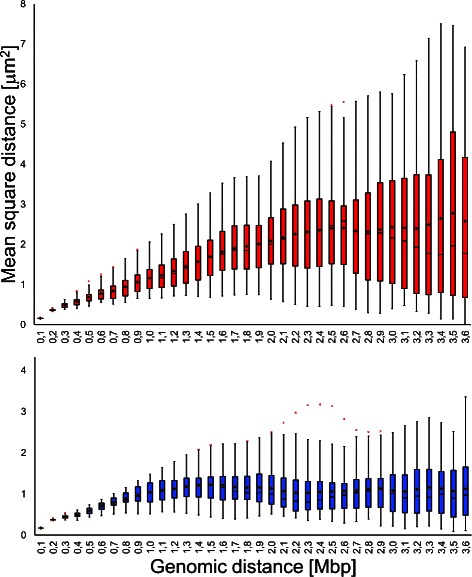
Structural differences. Boxplots from 40 results of the type in Fig. 4, obtained through the same parameter set. Top: highly expressed domain. Bottom: poorly expressed domain. The boxes include the second and third quartiles; the whiskers extend for (at most) 1.5 times the interquartile range above the 3^*rd*^ and below the 2^*nd*^ quartiles. Cross marks: extreme outliers; Diamond marks: mean values

With these fixed initial parameters, we run repeatedly the algorithm on the same data set to produce many configurations with comparable values of data fit, that is, with nearly equal (high) compatibility with the Hi-C data. From a two-class classification of the different configurations, we identified the basic types exemplified in Fig. 4, top panels (see also Additional files 5, 6 and 7). It is easy to see that the configuration on the left is less packed than the one on the right. To check the validity of these results, we used experimental data relative to GM06990, the human lymphoblastoid B cell line used for the Hi-C experiments that produced our data. Data from the ENCODE database were explored using the UCDS genome browser [45, 46]. We analyzed the tract of chromosome 1 used for the experiment, and selected two genomic regions. The first, encompassing 3.5 Mbp, spans from *q*=153.3 Mbp to *q*=156.8 Mbp, and is rich in genes, strongly sensitive to DNaseI, highly expressed (high level of Tanscription Factor Binding Sites) and with a high content of H3K4, which is a histone modification associated with highly expressed DNA. The second region, spanning from *q*=162 Mbp to *q*=165.5 Mbp, has a low gene density, is more resistant to DNaseI digestion, has low CTCF binding and low H3K4 level of methylation.^2^ The highly expressed domains are known to be much less packed than the domains poor in genes or with low transcriptional activity [47]. To verify the existence of this property in our results, following [48], we compare the genomic distances between pairs of loci with their Euclidean distance. The result is shown in the plots in Fig. 4, bottom panels, which represent the mean-square Euclidean distance between pairs of loci in the two stretches, as a function of their genomic distance. In the configuration shown on the left, the highly expressed domain is actually spread on a larger distance than the poorly expressed domain; in the configuration on the right, conversely, both domains occupy a small volume. This unexpected result could either depend on insufficient constraints, or capture real configurations assumed in some of the cells. In any case, Fig. 5 shows the boxplots for the two stretches, summarising 40 different results obtained using the same parameters (Additional files 5, 6, 7). Apparently, the two stretches show a substantially different statistical behaviour, and the poorly expressed region normally occupies much less space than the highly expressed region, although their genomic spans are nearly the same. These first tests thus demonstrate the biological plausibility of our results. The analysis of larger data sets, possibily using different cell types, will enable further refinement and confirmation of the validity of our method.

## Conclusion

We propose a new approach to estimate chromatin configurations from contact frequency data. The novelties introduced are a modified bead-chain model evolved by quaternion operators, and a data-fit function that does not require to translate frequencies into distances. The 3D structure can be estimated by applying our algorithm recursively at different resolutions. In order to keep the model compliant with known physical and biological features, any prior information available must be translated into geometrical constraints.

Our first results from real Hi-C data show that the configurations obtained are compatible with biological information that has not been introduced in the problem. Indeed, the geometrical constraints we introduce are uniform along the chain, so the structural differences only depend on data. Thus, we demonstrated that structurally different regions in our reconstructions highly correlate with functionally different regions as known from literature and genomic repositories.

Besides extending the experimentation to further data and target features, our future activity will deal with the optimisation of our code, in order to help the choice of the most appropriate parameters, include an explicit treatment of data biases, along with all the available biological knowledge, and allow structure estimation for larger and larger genomic regions.

## Endnotes

^1^ http://www.geometrictools.com/Documentation/RotationIssues.pdf (last additions. accessed: 2015, May 5^*th*^).

^2^ http://genome.ucsc.edu/ENCODE/ (last accessed: 2015, April 28^*th*^).

## Additional files

Additional file 1
**Basics on quaternion algebra.**

Additional file 2
**Python code for structure reconstruction at two levels of resolution (in this version, the relevant diagonal blocks must be provided as input).**

Additional file 3
**292** ***×*** **292 contact frequency matrix used to run the experiments reported in this paper.**

Additional file 4
**Bounds of the topological domains used to partition the original matrix.**

Additional file 5
**Plots of the 40 outputs used to build Fig.** 5 **(measurements in nm).**

Additional file 6
**Coordinates of all the 100-kbp beads for the 40 configurations shown in Additional file** 5 **(measurements in nm).**

Additional file 7
**Grapher file (For Mac OSX 10.9.5) showing the 3D structure of the reconstructed chain (in this example, Configuration1 from Additional file** 6 **.** To display other configurations, the coordinates in the 5 point sets can be replaced by the corresponding data).
