# Supplementary material for: Inferring 3D chromatin structure using a multiscale approach based on quaternions
Source: BMC Bioinformatics. 2015 Jul 29;16:234. doi: 10.1186/s12859-015-0667-0 (PMC4518643; doi:10.1186/s12859-015-0667-0)
Supplement: Additional file 1 — Basics on quaternion algebra. [file 12859_2015_667_MOESM1_ESM.pdf]

Inferring 3D chromatin structure using a multiscale approach based on quaternions
Supplementary Material

Claudia Caudai, Emanuele Salerno, Monica Zoppè, and Anna Tonazzini

Here we report an essential account on basic characteristics of quaternions. Further details on quaternion algebra can be found in [35]. Let us consider the following extended concept of a complex number:

$$\mathbf{q} = q_0 + q_1 i + q_2 j + q_3 k \tag{1}$$

where q_0, q_1, q_2 and q_3 are real numbers, and the imaginary units i, j and k obey the following fundamental formulas, which define the product of quaternions

$$\begin{array}{c} i^2 = j^2 = k^2 = -1 \\ ij = -ji = k \\ jk = -kj = i \\ ki = -ik = j \end{array} \tag{2}$$

If $q_0 = 0$, $\mathbf{q}$ is called a *pure quaternion*, and is the analogous of an $\mathbb{R}^3$ vector $(q_0, q_1, q_2)^T$. The conjugate, norm, and inverse of $\mathbf{q}$, respectively, are defined as

$$\bar{\mathbf{q}} = q_0 - q_1 i - q_2 j - q_3 k \tag{3}$$

$$||\mathbf{q}|| = \sqrt{\bar{\mathbf{q}}\mathbf{q}} = \sqrt{q_0^2 + q_1^2 + q_2^2 + q_3^2} \tag{4}$$

$$\mathbf{q}^{-1} = \frac{\bar{\mathbf{q}}}{||\mathbf{q}||^2} \tag{5}$$

Quaternions with $||\mathbf{q}|| = 1$ are called *unit quaternions.* Unlike real or complex products, the product of quaternions is not commutative. The rotation of a vector $(v_x, v_y, v_z)^T$, represented as the pure quaternion $\mathbf{v} = v_x i + v_y j + v_z k$, of an angle θ around a direction identified by the unit pure quaternion $\mathbf{u} = (u_x, u_y, u_z)^T$, can be expressed through the map $\mathbf{v} \to \mathbf{R_q}(\mathbf{v}) = \mathbf{q}\mathbf{v}\bar{\mathbf{q}}$, where $\mathbf{q}$ is the unit quaternion

$$\mathbf{q} = cos\frac{\theta}{2} + sin\frac{\theta}{2}u_x i + sin\frac{\theta}{2}u_y j + sin\frac{\theta}{2}u_z k \tag{6}$$

The composition of two rotations is simply obtained through the product of the corresponding quaternions: $\mathbf{R_p}(\mathbf{R_q}(\mathbf{v})) = \mathbf{R_{pq}}(\mathbf{v})$. Note that, as the quaternion product is non-commutative, it is $\mathbf{R_{pq}} \neq \mathbf{R_{qp}}$. Also, since quaternions $\mathbf{q}$ and $-\mathbf{q}$ give the same rotation (changing the sign of $\mathbf{q}$ is increasing θ of 2π), it is $\mathbf{R_q} = \mathbf{R_{-q}}$.
