# Supplementary material for: Inferring 3D chromatin structure using a multiscale approach based on quaternions
Source: BMC Bioinformatics. 2015 Jul 29;16:234. doi: 10.1186/s12859-015-0667-0 (PMC4518643; doi:10.1186/s12859-015-0667-0)

Inferring 3D chromatin structure using a multiscale approach based on quaternions – Supplementary Material

Claudia Caudai, Emanuele Salerno, Monica Zoppè and Anna Tonazzini

Data for a 30 Mbp stretch of Chromosome 1, long arm, from lymphoblastoid cell line GM06990 were retrieved for both the Hi-C [8] and the expression experiments (ENCODE). Two stretches of about 3.5 Mbp were selected as representative of, respectively, highly and poorly expressed genomic regions (see figure below):
Highly expressed: from q = 153.3 Mbp to q = 156.8 Mbp,
Poorly expressed: from q = 162 Mbp to q = 165.5 Mbp.

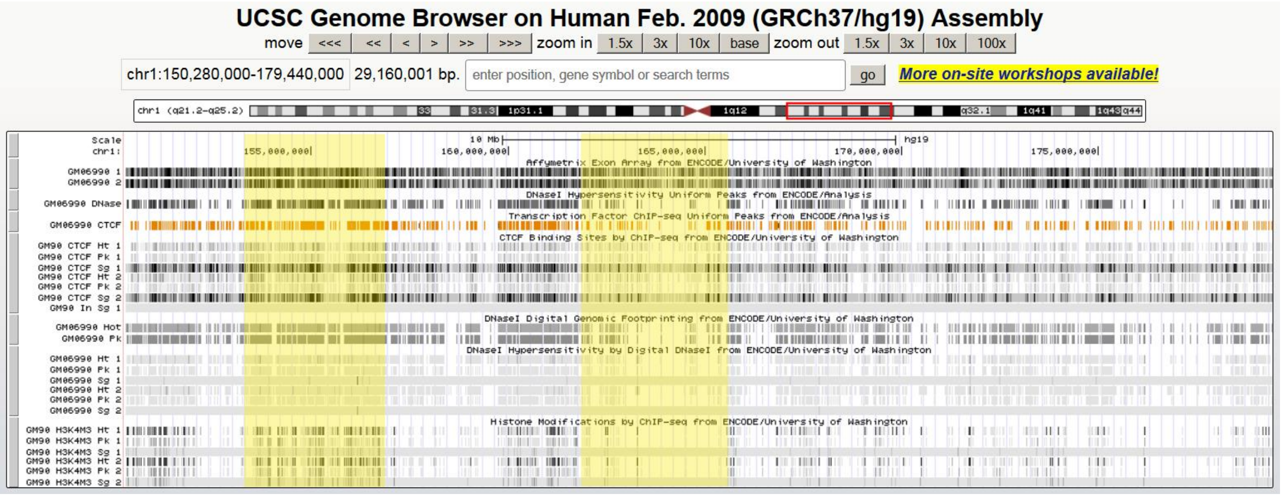

Applying our algorithm we obtained 40 configurations. For each configuration, we calculated the mean-square Euclidean distances between pairs of loci as functions of their genomic distance in the high and low expression regions (see Fig. 4 of main text). The ratio ρ between the integral of the functions obtained for the two regions is reported in Table 1.

Two clusters were identified, defined by the threshold value $\rho = 2$. Those with $\rho > 2$ (meaning that the span of the highly expressed region is at least twice as much as the poorly expressed counterpart) show a peculiar pear-like shape, clearly recognizable in the plots below.

Table 1: Ratios ρ of the 40 configurations

Configuration	Ratio ρ
1	1.99
2	1.03
3	1.13
4	1.72
**5**	**2.51**
6	0.89
**7**	**5.66**
**8**	**3.36**
9	1.19
10	0.85
11	1.15
12	1.89
**13**	**2.41**
**14**	**2.92**
**15**	**3.28**
16	1.37
**17**	**3.03**
**18**	**2.46**
19	0.64
20	0.47
21	0.69
22	1.11
23	0.63
**24**	**3.11**
25	1.62
**26**	**4.02**
**27**	**2.72**
28	1.49
29	1.34
**30**	**3.85**
31	1.54
32	1.82
33	0.54
**34**	**2.26**
35	1.73
**36**	**2.72**
37	1.19
**38**	**2.49**
39	0.92
**40**	**4.05**

Final configurations with ρ > 2, “pear like” conformation (5-7-8-13-14-15-17-18-24-26-27-30-34-36-38-40):

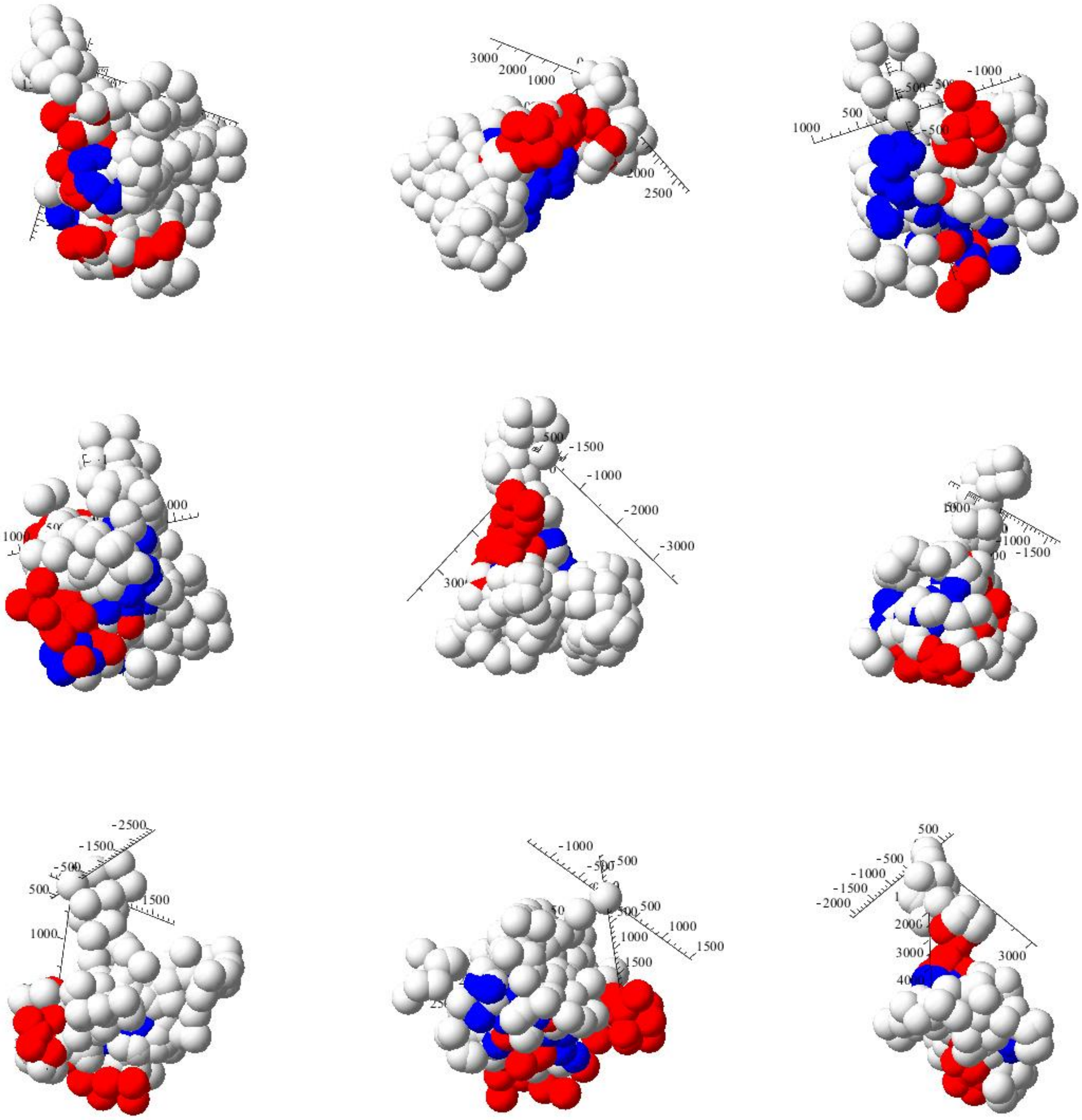

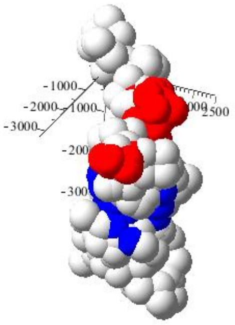
-1000
-2000
-3000
2500

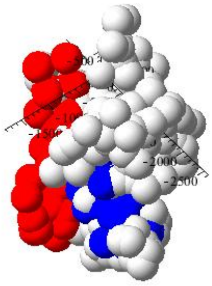
-2000
-2500

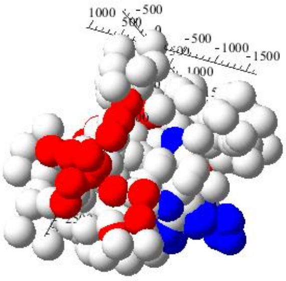
1000
-500
-500
-1000
-1500
1000

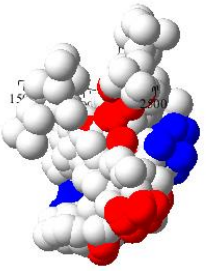

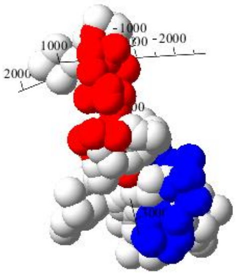
-1000
-2000
1000
2000

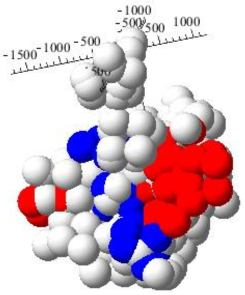
-1000
-1500
-1000
-500
1000

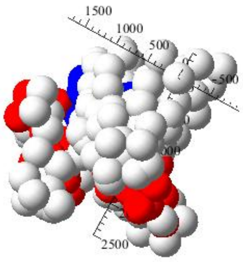
1500
1000
500
-500
2500

Final configurations with ρ < 2, “globular” conformation (1-2-3-4-6-9-10-11-12-16-19-20-21-22-23-25-28-29-31-32-33-35-37-39):

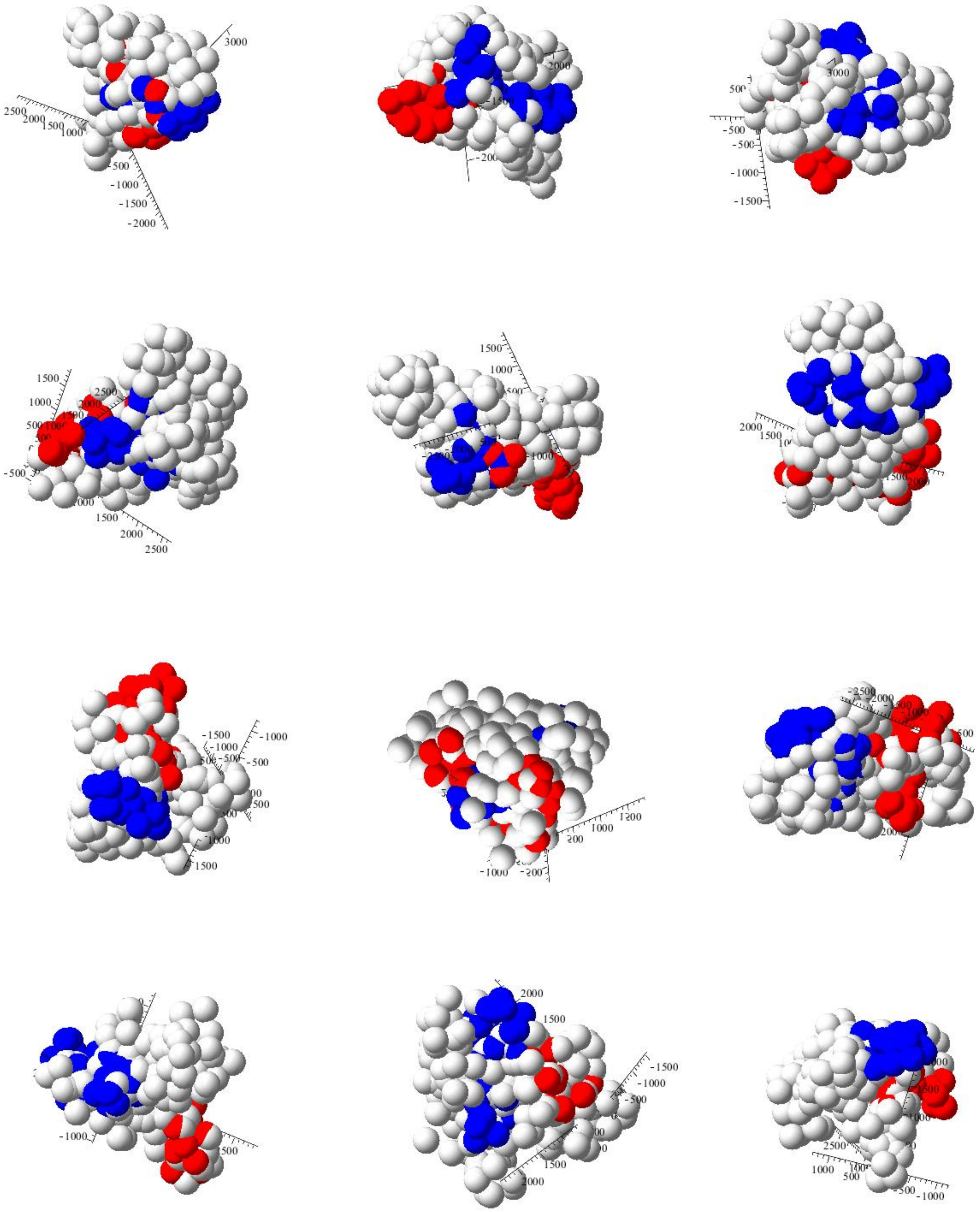

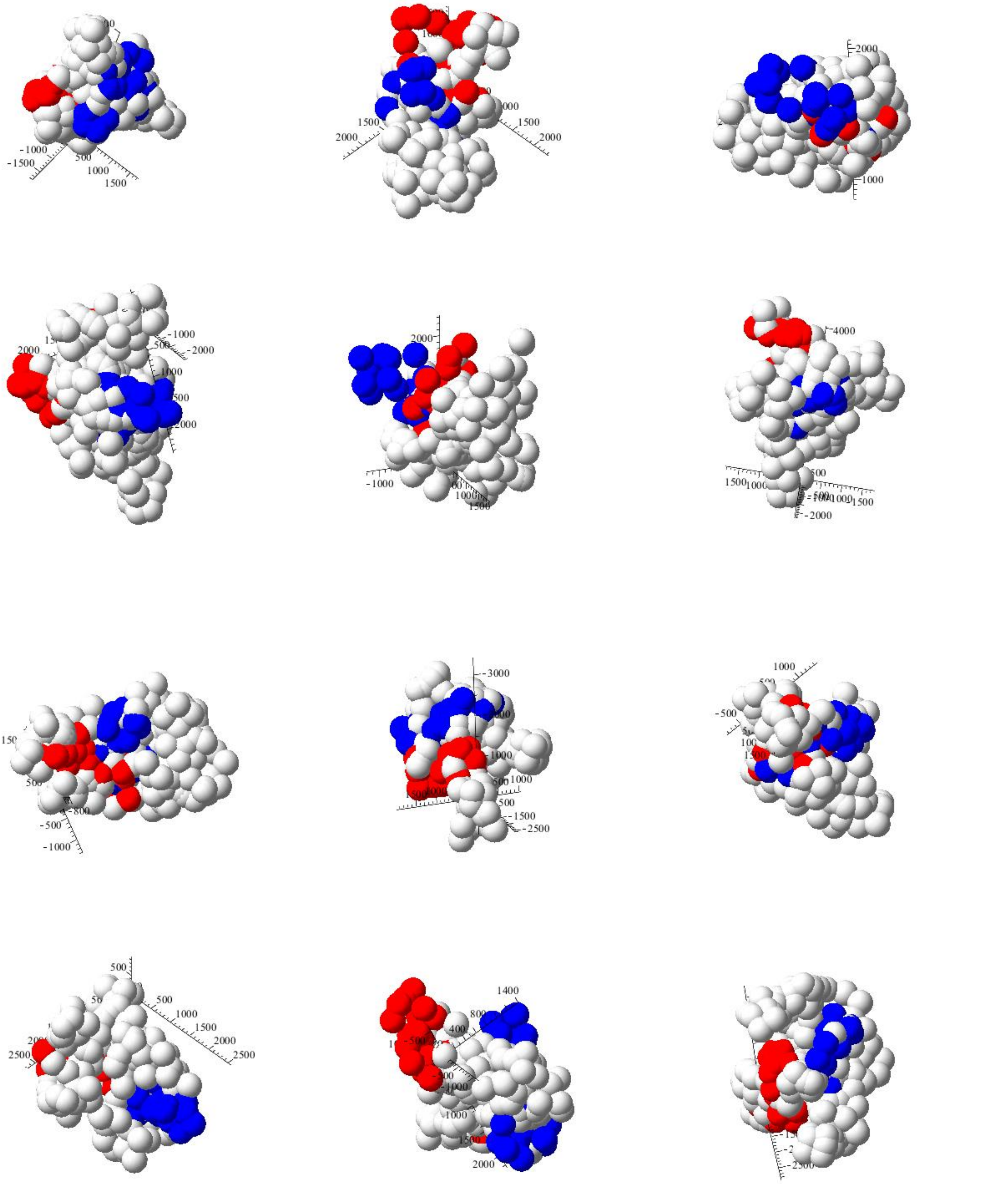

Supplement: Additional file 5 — Plots of the 40 outputs used to build Fig. 5 (measurements in nm). [file 12859_2015_667_MOESM5_ESM.pdf]
